# Arthroscopic Bankart repair using trans-glenoid double-loaded grand knots versus double-loaded suture anchors; is there a difference? a randomized controlled study

**DOI:** 10.1186/s12891-025-08477-3

**Published:** 2025-03-21

**Authors:** Amr Samir Rashwan, Al-Qassem Amin, Sherif Hamdy Zawam, Alaa Mohy-Eldin Soliman, Mahmoud El-Desouky

**Affiliations:** 1 Department of Trauma and Orthopedics, Faculty of Medicine, Cairo University, Cairo, Egypt; 2 Al-Helal Hospital, Cairo, Egypt

**Keywords:** Shoulder dislocation, Bankart, Double-loaded anchor, Grand knot, Trans-glenoid

## Abstract

**Background:**

Anatomical repair of Bankart lesions and restoring the tension of the antero-inferior capsulo-labral complex is the optimum method of surgical treatment with a variety of fixation methods including suture anchors and trans-glenoid sutures. Grand knot technique is a modification of the trans-glenoid sutures technique that can be an alternative to double-loaded suture anchors with a lower cost. We aimed to compare the outcomes and complications of both techniques.

**Methods:**

This is a randomized controlled study that was conducted on 200 patients with recurrent anterior glenohumeral dislocation, of whom 170 patients completed at least a three-year follow-up period. Arthroscopic Bankart repair using two double-loaded knotted suture anchors was performed in 78 cases (Group A) while repair was done using two trans-glenoid grand knots in other 92 cases (Group B). Patients were evaluated in terms of range of motion, functional scores (Constant, Rowe, and ASES), and complication rate.

**Results:**

The mean operative time was significantly longer in Group B (87.7 ± 24) minutes compared to Group A (61.2 ± 28.1) minutes (*P* = 0.002). No statistically significant difference was found between both groups regarding postoperative external rotation range of adducted arm, functional scores, and rate of recurrence. Only forward flexion and external rotation of abducted arm were significantly better in Group A (*P* = 0.005 and < 0.001 respectively).

**Conclusion:**

Trans-glenoid double-loaded grand knot technique is an alternative surgical option for the treatment of Bankart lesions with comparable results to double-loaded anchors regarding the functional outcomes and failure rates.

**Clinical Trial Registration (Retrospectively registered):**

Registration number: NCT06394609 28-4-2024.

**Supplementary Information:**

The online version contains supplementary material available at 10.1186/s12891-025-08477-3.

## Introduction

The glenohumeral joint is the most frequently dislocated joint representing more than 50% of all joint dislocations [1]. Recurrence is the major complication following traumatic anterior shoulder dislocation, as it accounts for an average of 70–90% in patients aged 20 to 40 years [2].

Traumatic anterior glenohumeral joint instability usually results in Bankart lesion which is an avulsion injury of the antero-inferior labrum [3]. Arthroscopic Bankart repair has been a widely accepted method for restoring anterior shoulder stability with comparable results to open repair techniques being less invasive, cosmetic, safe, with shorter time of surgery, improved range of motion, and less post-operative pain [4].

There have been remarkable changes and progress in arthroscopic Bankart repair surgeries over the past years regarding the methods and implants used for fixation. Metallic, biodegradable, bio-composite, and finally all-suture suture anchors have been used [5, 6].

Trans-glenoid pullout suture technique is a good alternative which can serve the same function as suture anchor techniques with lower costs [7]. Double-loaded single-row repair using either double-loaded anchors or double-loaded grand knots can allow a strong labral repair with fewer number of suture anchors or trans-glenoid tunnels needed [8].

In the current study, we used the Grand knot technique which is a suture block that rests on the posterior glenoid surface with two strands of sliding OrthoCord passing through it. We hypothesized that the use of double-loaded grand knots would show similar outcomes compared to double-loaded knotted suture anchors with lower cost. We, therefore, aimed to compare both techniques regarding their clinical outcome, and complications rate.

## Methods

This was a prospective randomized controlled study that was conducted in our Department from December 2017 to April 2023. We included skeletally mature patients with single or multiple episodes of shoulder instability suffering from Bankart lesion or its variants as ALPSA or Perthes lesions. Two hundred patients were enrolled in this study, after excluding patients with significant glenoid or humeral bone loss (bony Bankart and or Hill Sachs lesions), uncontrolled epileptic fits, and those with multi-directional instability showing signs of hyperlaxity. Included patients were evaluated by detailed history taking, thorough clinical examination, and imaging including CT and MRI of the affected shoulder.

**Fig. 1:**
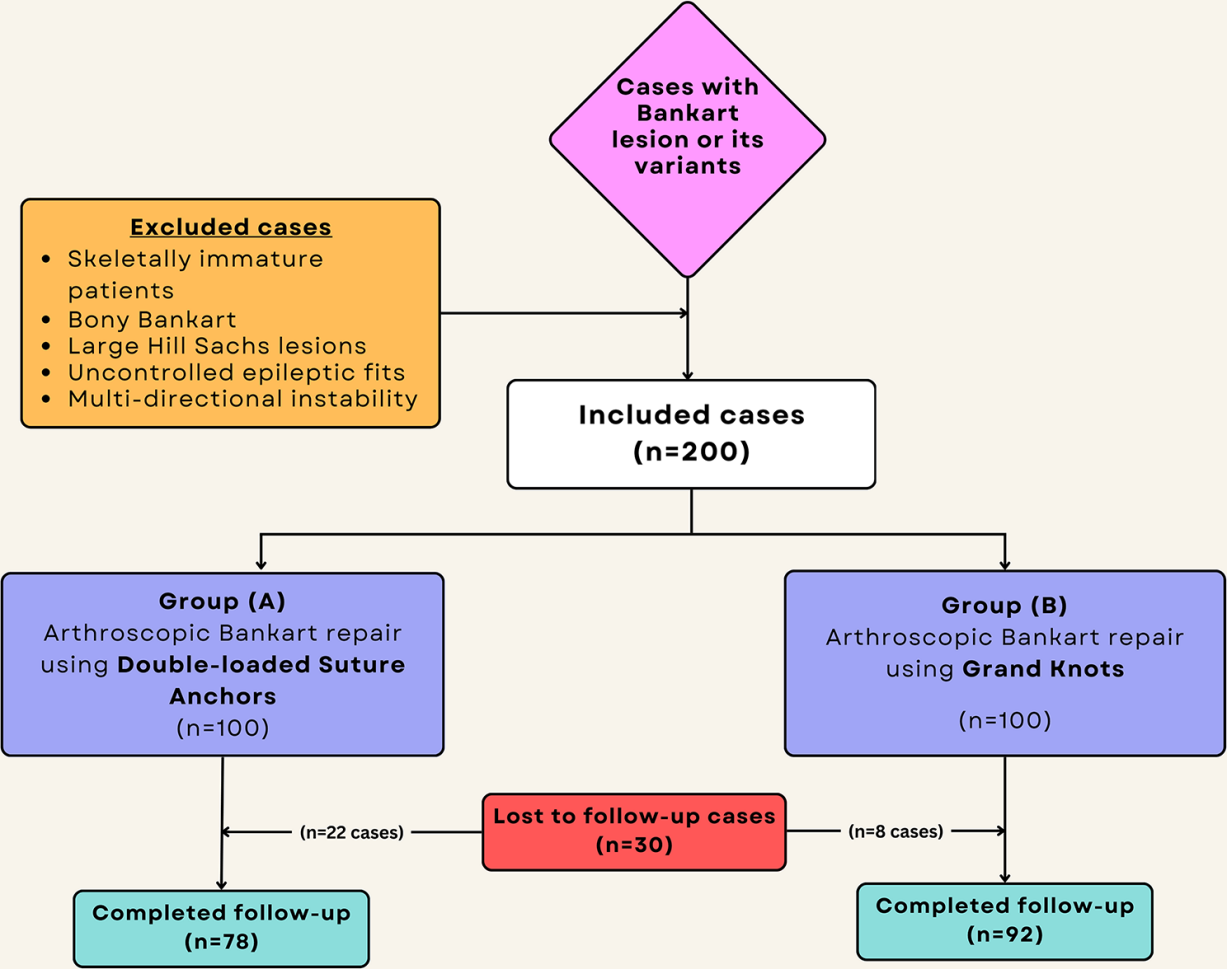
Flow chart of included cases

The patients were divided into two equal groups after being randomized through the closed envelope technique using cards with numbers from one to 200. Each time, one of these cards was picked, and accordingly, cases with odd numbers (100 patients) were allocated to Group-A to whom arthroscopic Bankart repair was done using two double-loaded suture anchors, and those with even numbers (100 patients) were allocated to Group-B to whom arthroscopic Bankart repair was done using two double-loaded grand knots. A total of 30 patients were lost to follow-up, leaving 170 patients (78 in Group-A and 92 in Group-B) at the end of the study who completed three years of follow-up (Fig. 1).

### Surgical procedure

All patients were operated upon in Beach chair position and under general anesthesia. After sterilization and draping, the posterior portal was used as a viewing portal. Then the anterior-inferior and the anterior-superior portals were established. Diagnostic arthroscopic examination was performed routinely.

**Fig. 2:**
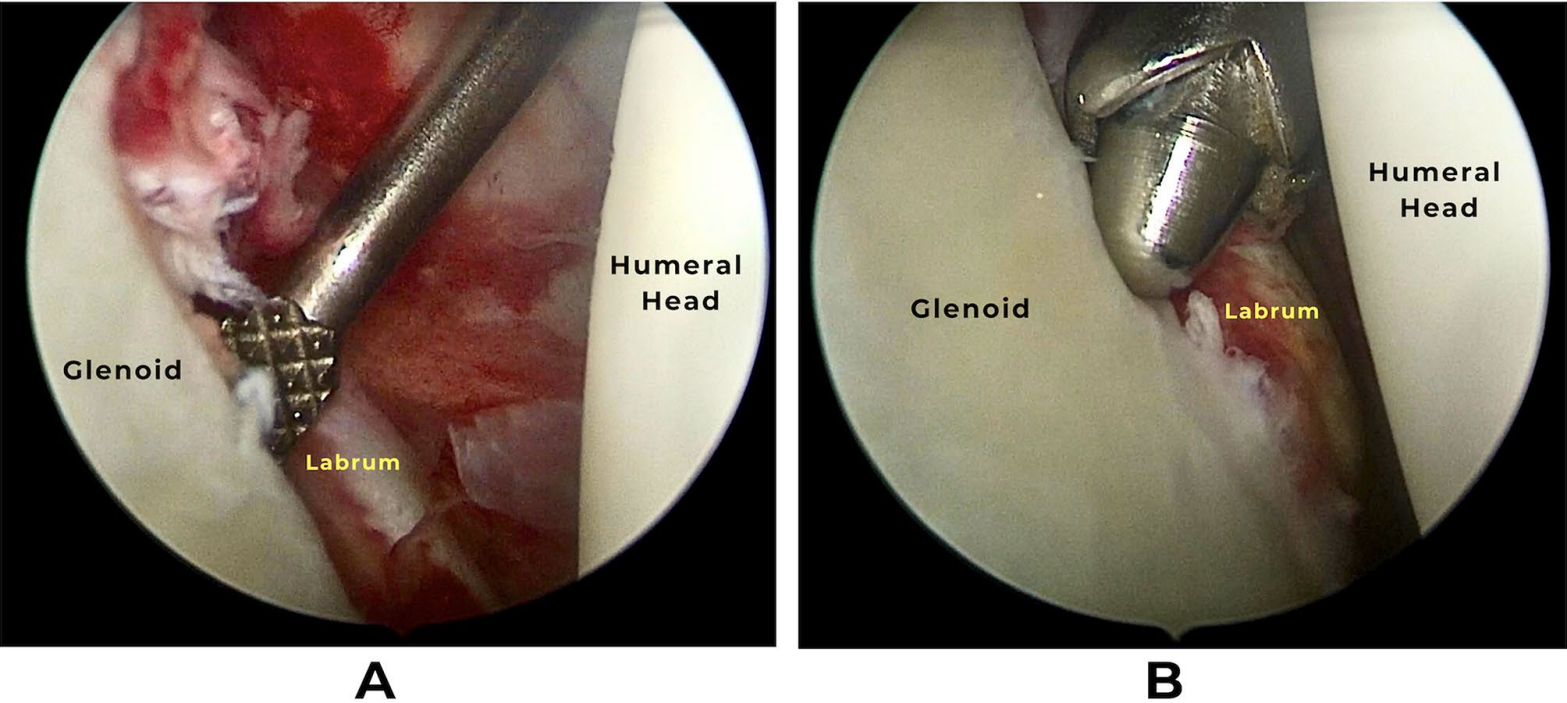
Arthroscopic view of the right shoulder showing the preparation for repair **(A**) Labrum release with arthroscopic elevator, **(B)** Debridement with motorized shaver

Adequate preparation for Bankart repair was carried out by capsulo-labral release medially till subscapularis muscle fibers and inferiorly as far as 6 o’clock using arthroscopic elevator. The exposed glenoid edge opposite to the labral lesion was debrided with a shaver and then a rasp to promote healing (Fig. 2).

### Double-loaded anchor method

**Fig. 3:**
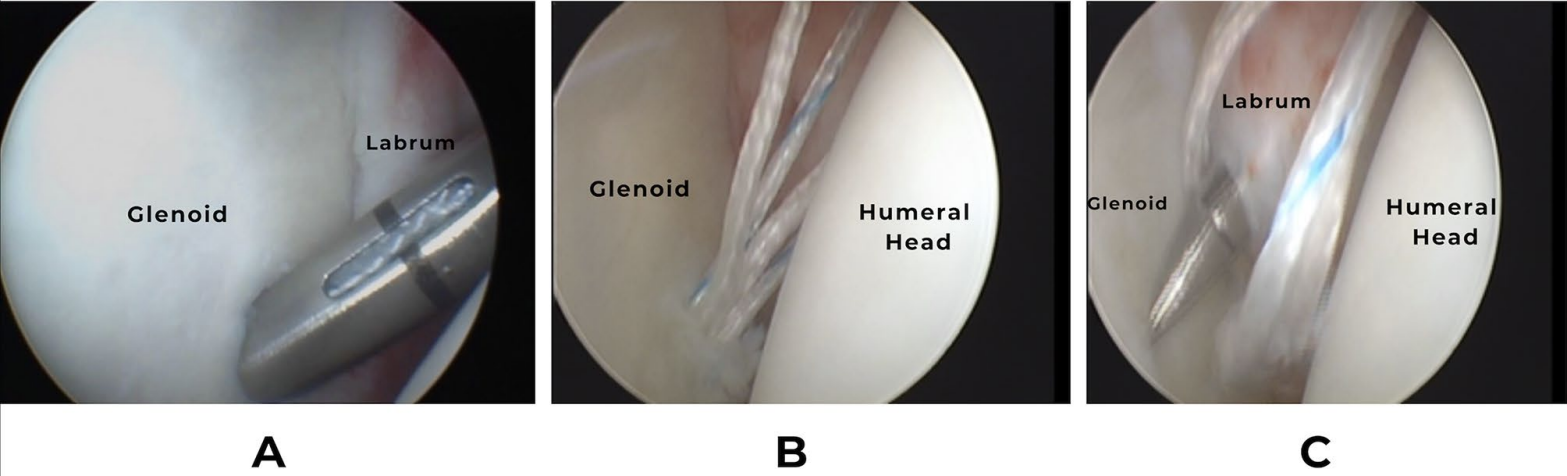
Arthroscopic view of the right shoulder showing the steps of Bankart repair with double-loaded anchors **(A)** Anchor insertion at 5 o’clock, 2 mm from anterior edge of the glenoid using sleeve, **(B)** Checking anchor position and hold, **(C)** A sharp tip suture retriever (Parrot Beak) passed through labral tissue

In Group-A patients, two double-loaded suture knotted anchors; FASTac-Arthrex (2.8 mm) or JuggerKnot-Zimmer-Biomet (2.9 mm) containing two differently-colored No.2 non-absorbable braided sutures were used. The first one was inserted at 5 o’clock position for the right shoulder and 7 o’clock position for the left shoulder. The anchor was inserted 2 mm from the anterior edge of the glenoid and angulated 45^ο^ on the glenoid surface (Fig. 3).

A sharp tip suture retriever (Parrot Beak) was passed through labral tissue below the anchor and one limb of the suture was retrieved for the first thread. A sliding knot with subsequent three locking half hitches were tied. The two tails of the completed knot were cut 5 mm above the knot with the arthroscopic scissors.

The same process was repeated for the second thread and for the second superior anchor which was placed at 4 or 8 o’clock position for the right or left shoulder respectively.

### Double-loaded grand knot technique

**Fig. 4:**
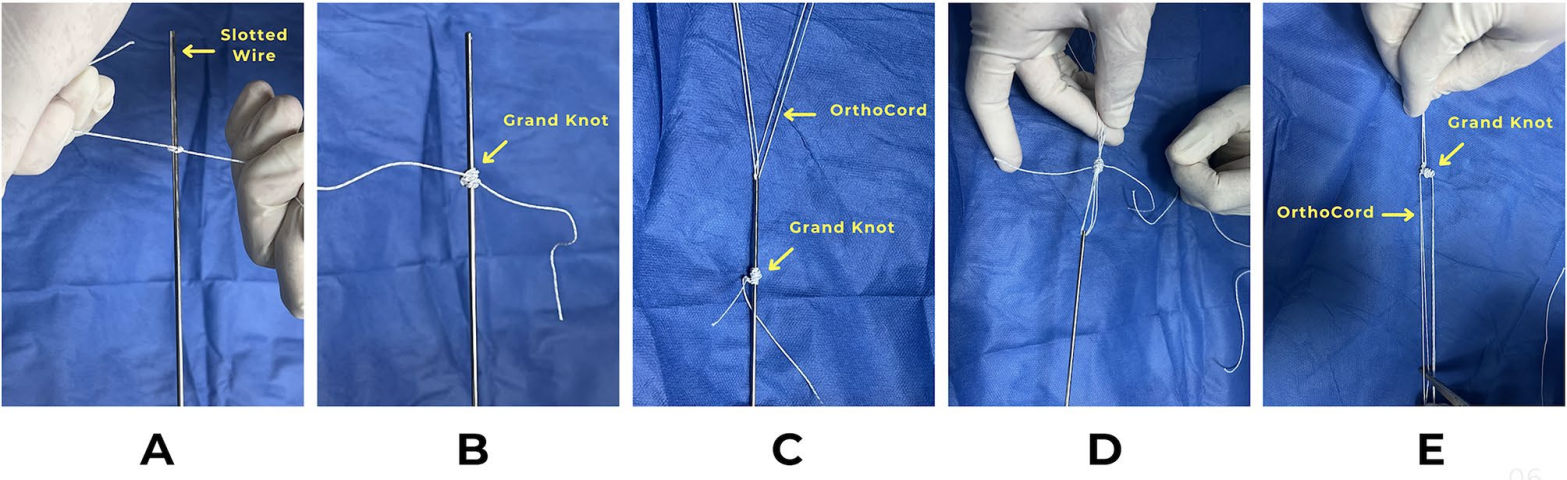
Steps of Grand knot preparation **(A)**, **(B)** Tying the suture block over a slotted guidewire, **(C)** Shuttling the OrthoCord wires through the slotted guide wire, **(D)** Passing the OrthoCord through the suture block, **(E)** The final construct of the Grand knot and the 2 pairs of sliding OrthoCord wires

In Group-B patients, two double-loaded grand knots were prepared with a suture block (#5 FiberWire Suture) hanging on the posterior aspect of the glenoid neck and two OrthoCord suture-wires (#2 FiberWire Suture) were passed through the suture block to slide within the drilled bony tunnels. The suture block was made of 20 repetitive knots each five were knotted in a different direction; regular or reverse (Fig. 4).

**Fig. 5:**
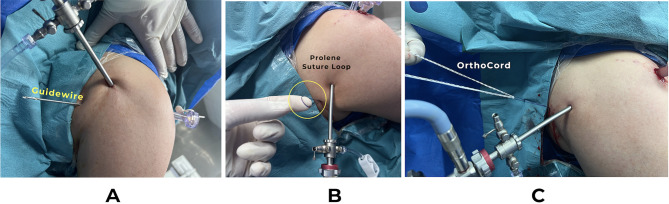
**(A)** A 2.4 mm slotted guidewire exiting posteriorly through the safe zone, **(B)** Prolene suture loop at the posterior exit after pulling the guidewire anteriorly, **(C)** The four ends of both OrthoCord wires passed through the prolene loop

A drill guide was held at 5 or 7 o’clock position for the right or left shoulder respectively, at the anterior edge of the glenoid and angulated 25^ο^ on the glenoid surface and less than 20^ο^ in the caudal direction. Then a 2.4 mm guidewire was introduced from anterior to exit posteriorly through the safe zone; 7–10 cm below the acromion and passed throughout the skin, where a one-cm skin incision was made over its exit point (Fig. 5).

Protecting the suprascapular nerve during this procedure requires precise placement of the entry point at the anterior glenoid edge, and avoiding the divergence of the guidewire from the glenoid surface more than 25^o^. Furthermore, we only used the power drill to pass the guidewire through the bony glenoid then a hammer for gentle passage through soft tissue to the skin posteriorly.

**Fig. 6:**
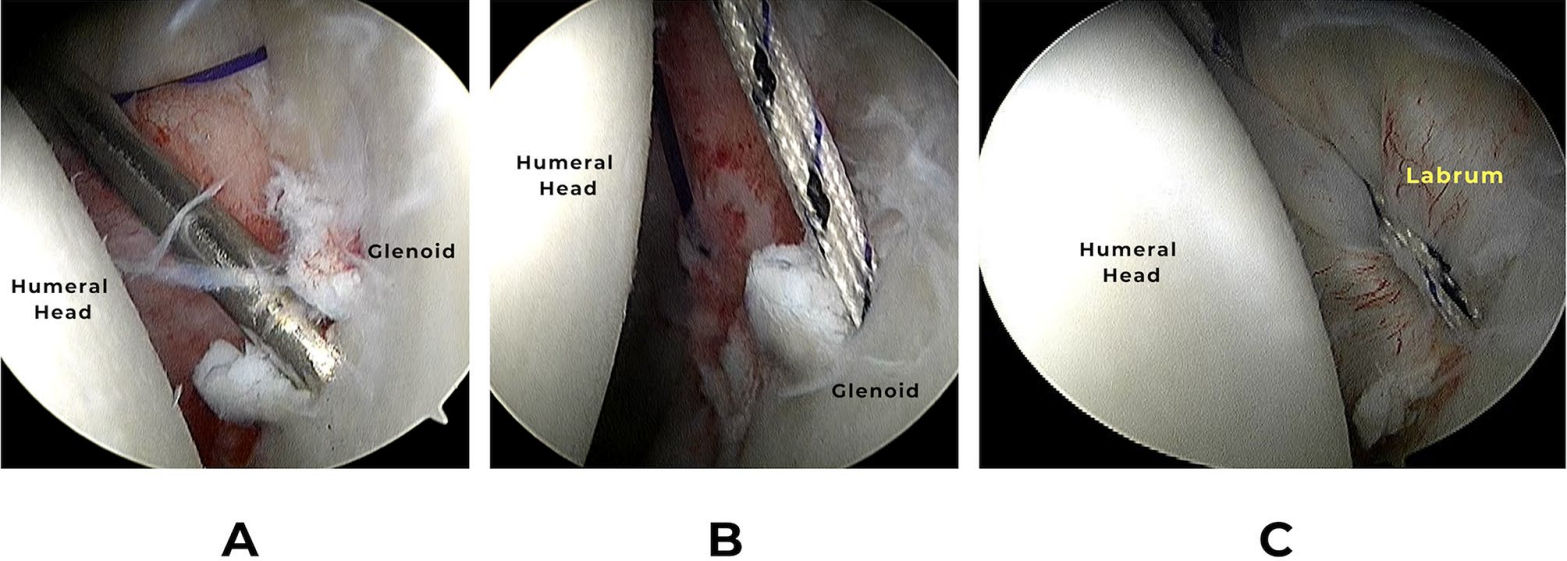
Arthroscopic view of the left shoulder showing the steps of repair with Grand knot **(A)** Introducing the guidewire, **(B)** The four ends of the Grand knot are pulled into the tunnel, **(C)** The final view after labral repair

**Fig. 7:**
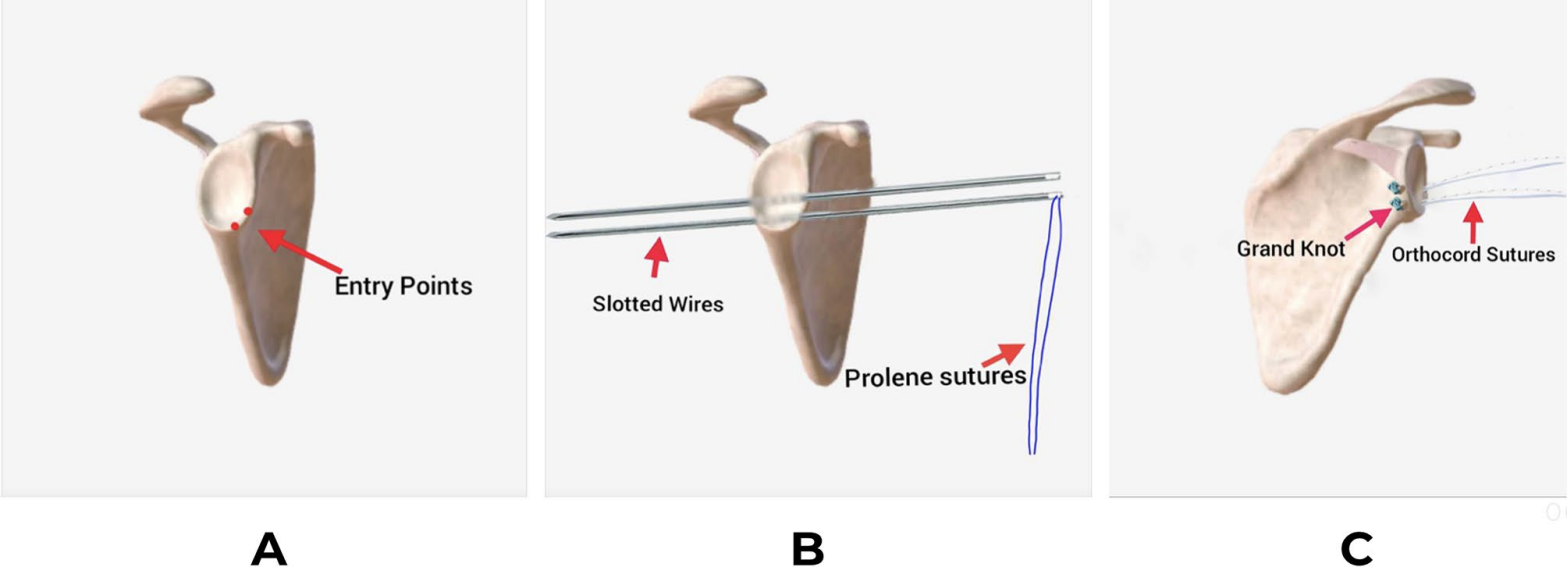
Diagrammatic view of the right shoulder showing the steps of the Grand Knot passage **(A)** Entry points of the slotted wires, **(B)** The slotted wires passing through the glenoid from anterior to posterior with the prolene sutures for later on shuttling, **(C)** Final view of the Grand Knot resting on the posterior glenoid surface and the OrthoCord sutures passing anteriorly

Using a T-handle, the guidewire was pulled out from its posterior exit together with No.1 prolene suture loop with its two ends coming out of the cannula anteriorly. The four ends of both OrthoCord wires (Sliding Sutures) of the grand knot were passed through the prolene loop, and they were retrieved anteriorly through the anterior cannula (Fig. 6) (Fig. 7). Before pulling the Grand Knot, a straight artery forceps was introduced through the posterior incision to dissect through the subcutaneous tissue and the muscles down to the posterior glenoid surface. Then the four OrthoCord wires were pulled until a click was felt which indicates that the Grand Knot was secured directly on the bone with no soft tissue interposition. The absence of posterior skin puckering confirms right placement of the knot.

Suture handling of each pair of the OrthoCord wires was done as the anchors’ group (Fig. 5). Then, the second grand knot was placed at 4 or 8 o’clock position for the right or left shoulder respectively.

### Physiotherapy and rehabilitation

The shoulder was immobilized in a broad arm sling for four weeks, and the patient was instructed to move his elbow and wrist freely and pendulum exercises were allowed. Then physiotherapy sessions were started, with passive and gentle active-assisted range of motion (ROM) exercises. Restoration of full active ROM, muscle strength, and neuromuscular control were carried out gradually. The return to work and sports was allowed according to the progression of each patient in the rehabilitation pathway.

The patients were followed at 2,4,6,8 and 12 weeks after surgery. Then they were evaluated during visits at 6, 12, and 24 months. After three years, the final outcome was assessed using Constant, Rowe, and ASES Scores.

### Statistical methods

Data was summarized using the mean and standard deviation or count and percentages. Comparisons were done using unpaired t-test, Mann-Whitney test, Fisher’s Exact test, or Logrank test. P-values < 0.05 were considered as statistically significant. SPSS 22 was used.

## Results

The mean age of patients included in this series was 31.7 ± 8.1 years (range:19–48) with 148 males (87.1%) and 22 females (12.9%). No significant statistical differences were detected when comparing the demographic characteristics except for the number of episodes of dislocation which was significantly higher in Group-B (*p* = 0.007) (Table 1).

**Table 1:** Demographic features of included cases and operative data

	Group A (Anchors group) (*n* = 78)	Group B (Grand knots group) (*n* = 92)	*P*-value
Age (years)	32.4 ± 7.1	31.2 ± 8.0	0.614
Sex			0.156
Male	71 (91.0%)	77 (83.7%)	
Female	7 (9.0%)	15 (16.3%)	
Athletes	32 (41.0%)	43 (46.7%)	0.455
Professional	9 (11.5%)	17 (18.5%)	
Recreational	23 (29.5%)	26 (28.3%)	
Operated side			0.163
Dominant	57 (73.1%)	58 (63.0%)	
Non-dominant	21 (26.9%)	34 (37.0%)	
Frequency of dislocation	5.4 (3–7)	12.4 (6–23)	0.007
First dislocation to surgery time (months)	10.5 (6–24)	24.1 (12–48)	0.082
Operative time (minutes)	61.2 ± 28.1	87.7 ± 24.0	0.002
Recurrence	1 (1.3%)	2 (2.2%)	1

The time interval between the first dislocation and surgery was comparable in both groups (from 6 to 48 months) with a 15.4 months mean-value. The mean operative time was longer in Group-B (87.7 ± 24.0 min) as compared to Group-A (61.2 ± 28.1 min) with a statistically significant difference (*p* = 0.002) (Table 1).

There was a significant increase in the postoperative range of forward flexion, and external rotation in adducted and abducted arm positions in both groups. There was also a statistically significant difference between both groups regarding the postoperative range of forward flexion and external rotation at 90° abduction as it was better in Group-A as compared to Group-B (p-value: 0.005 and < 0.001 respectively) while there was no statistically significant difference regarding the postoperative range of external rotation with arms adducted (*p* = 0.450) (Table 2).

**Table 2:** Range of motion in the operated shoulder

Range of motion	Forward flexion (°)			External rotation with arm adducted (°)			External Rotation with arm abducted 90^o^ (°)		
	Group A (Anchors group) (*n* = 78)	Group B (Grand knots group) (*n* = 92)	*P*-value	Group A (Anchors group) (*n* = 78)	Group B (Grand knots group) (*n* = 92)	*P*-value	Group A (Anchors group) (*n* = 78)	Group B (Grand knots group) (*n* = 92)	*P*-value
Preoperative	161.5 ± 25.3	136.5 ± 31.8	< 0.001	59.1 ± 10.2	47.5 ± 12.1	0.014	83.8 ± 15	60.3 ± 9.7	< 0.001
Postoperative	176.2 ± 4.5	167.3 ± 12.4	0.005	63.5 ± 8.4	59.8 ± 19.9	0.450	89.0 ± 10.4	75.8 ± 11.4	< 0.001
P-value	0.013	< 0.001		0.032	0.016		0.014	< 0.001	

There were no statistically significant differences between both groups regarding the mean postoperative Rowe, Constant, and ASES scores (*p* = 0.188, 0.931, and 0.323 respectively) (Table 3).

**Table 3:** Functional scores in operated shoulder at final follow-up

Functional scores	Group A (Anchors group) (*n* = 78)	Group B (Grand knots group) (*n* = 92)	*P*-value
Rowe score	81 ± 20	89 ± 17	0.188
Constant score	82 ± 6	81 ± 11	0.931
ASES score	90 ± 8	92 ± 8	0.323

When evaluating the cost of used implants or sutures in both groups, we found that it was around 640 USD for the two anchors (320 each) in Group-A, compared to 70 USD for the two sutures in Group-B.

Regarding the return to athletic activities, 75 patients in both groups (32 in Group-A and 43 in Group-B) were either professional or recreational athletes. Sixty-two patients (82.7%) returned to the same level of athletic activity by 6 months. Other seven patients (9.3%) had a late return to athletic activity before one year while six patients (8%) had to quit their participation in athletic activities following surgery. There were no statistically significant differences between both groups regarding the return to athletic activities.

Recurrence was experienced following major trauma by 1 patient (1.3%) in Group-A at 13 months compared to two patients (2.2%) in Group-B) at eight-, and 12 months post-surgery. Laterjet procedure was performed in all these cases. There was no statistically significant difference in comparison between the two groups (*p* = 1.000). None of Group-B patients experienced suprascapular nerve injury.

## Discussion

Recurrent shoulder dislocation is a common problem affecting 1.7% of the population [9]. The main aim of treatment is to prevent recurrence with a safe technique with lower complication rate. There have been several surgical techniques with different outcomes regarding the functional outcome, rate of recurrence, and associated complications [10].

Since the introduction of the concept of Bankart lesions in the anteroinferior glenoid labrum as a potential cause of recurrent anterior dislocation in 1923, several procedures were described to approach this problem [3, 5]. Arthroscopic Bankart repair techniques have gained increasing popularity, with almost equal success rate as open repair [4]. Over the past 40 years, advances in anchors and sutures manufacture have contributed to better outcomes and higher success rates [5, 6].

We compared the results of 78 cases who underwent arthroscopic Bankart repair using two double-loaded suture anchors to 92 patients using two trans-glenoid double-loaded grand knots. The mean age of included patients was 31.7 years. The mean time between the first dislocation and surgery was 15.4 months. The mean operative time in the Grand Knot group (87.7 min) was significantly longer than the Suture anchor group (61.2 min).

Although a relatively better postoperative ROM was obtained in the Anchor group, the results were statistically significant in forward flexion and external rotation with the arm abducted, and did not achieve a statistically significant difference regarding external rotation with the arm adducted. Functional outcome assessments with Rowe, Constant, and ASES scores as well as the recurrence rate revealed no statistically significant difference between both groups.

Several studies have demonstrated significant improvements in clinical outcomes following arthroscopic labral repair, whether utilizing suture anchors or trans-glenoid sutures. These techniques are also associated with a notable reduction in postoperative re-dislocation rates (Table 4) [11–17].

**Table 4:** Comparison with the data of other studies

Study	Procedure	Cases	Age (years)	Frequency of dislocations	Interval between initial dislocation & surgery (months)	Follow-up (months)	ASES		Rowe		ROM	Complications	
							Pre	Post	Pre	Post		Re-dislocation	Other complications
Kim et al. [11]	Arthroscopic Bankart repair using Double-loaded suture anchors	45	23.7 (14–47)	6.8	47.9 (6-218)	28 (24–45)	67.3	96.9	68.7	96.8	Forward flexion 178.5^o^ External rotation 56.2^o^ (significantly decreased) Internal rotation 62.3^o^	4 (8.9%)	NR
Chalmers et al. [12]	Arthroscopic Bankart repair using Double-loaded suture anchors	30	30 (12–51)	12	56	80.4 (24–137)	NR	90	NR	NR	NR	4 (13%)	NR
Pagnani et al. [13]	Arthroscopic Bankart repair using trans-glenoid sutures	37	24.6 (15–45)	2–30	NR	67.2 (48–120)	NR	NR	NR	> 90 in 22 cases	Mean loss of forward flexion = 2° Mean loss of External rotation = 4° at both 0° and 90° of scapular plane elevation (Both were statistically significant)	7 (19%)	Synovial cyst: 1 case (2.7%)
Ekelund et al. [14]	Arthroscopic Bankart repair using trans-glenoid sutures	24	26 (16–46)	NR	6-180	37 25–51	NR	NR	NR	89	loss of External rotation 5^o^	2 (8%)	Transient suprascapular nerve injury: 1 case (4.2%)
van Oostveen et al. [15]	Arthroscopic Bankart repair using trans-glenoid sutures	165	27.5	NR	NR	80	NR	NR	NR	NR	NR	57 (34%)	Transient suprascapular nerve injury: 2 cases (1.2%)
	Arthroscopic Bankart repair using suture anchors	81	26.6	NR	NR	27	NR	NR	NR	NR	NR	7 (8.7%)	NR
Zaffagnini et al. [16]	Arthroscopic Bankart repair using trans-glenoid sutures	49	35	4	NR	164.4	NR	NR	NR	85	NR	6 (12.5%)	Severe osteoarthritis: 2 (4%)
	Open Bankart repair using trans-glenoid sutures	33	38	4	NR	188.4	NR	NR	NR	83.2	NR	3 (9%)	Severe osteoarthritis: 2 (6%)
Kandziora et al. [17]	Arthroscopic Bankart repair using trans-glenoid sutures	108	26.6	NR	NR	54 (24–95)	NR	NR	35	68.3	40 patients (37%) > > No External rotation loss 56 patients (51.9%) > > External rotation loss < 10^o^ 12 patients (11.1%) > > External rotation loss > 10^o^	35 (32.4%)	Transient suprascapular nerve injury: 3 cases (2.8%)
	Arthroscopic Bankart repair using suture anchors	55	28	NR	NR	38.4 (24–60)	NR	NR	35.4	84.6	19 patients (34.5%) > > No External rotation loss 28 patients (50.9%%) > > External rotation loss < 10^o^ 8 patients (14.6%) > > External rotation loss > 10^o^	9 (16.4%)	NR
Our Study	Arthroscopic Bankart repair using Double-loaded suture anchors	78	32.4 (19–48)	5.4 (3–7)	10.5 (6–24)	36.4 (36–41)	NR	90	NR	81	Forward flexion changed from 161.5 to 176.2^o^ External rotation with adducted arm changed from 59.1 to 63.5^o^ External rotation with abducted arm changed from 83.8 to 89.0^o^	1 (1.3%)	NR
	Arthroscopic Bankart repair using Grand knots (trans-glenoid)	92	31.2 (19–45)	12.4 (6–23)	24.1 (12–48)	36.7 (36–47)	NR	92	NR	89	Forward flexion changed from 136.5 to 167.3^o^ External rotation with adducted arm changed from 47.5 to 59.8^o^ External rotation with abducted arm changed from 60.3 to 75.8^o^	2 (2.2%)	NR

NR: Not reported

Among the largest comparative series is the retrospective study by Van Oostveen et al., which included 246 cases: 165 patients underwent labral repair using trans-glenoid sutures, and 81 were treated with suture anchors. In comparison, our study—comprising 170 cases (92 in the trans-glenoid group and 78 in the suture anchor group)—was conducted prospectively, which reduces the chances of bias [15].

The recurrence rate in the Van Oostveen et al. series was reported as 34% (57 cases) in the trans-glenoid group, aligning with similar findings by Kandziora et al., who observed a recurrence rate of 32.4% (35 cases) in their trans-glenoid group [15, 17]. Notably, other referenced studies reported recurrence rates of less than 10% (Table 4) [11–17]. In contrast, our study demonstrated significantly lower recurrence rates: 1.3% in the suture anchor group and 2.2% in the trans-glenoid group.

However, the trans-glenoid suture technique for arthroscopic Bankart repair is associated with potential complications, such as suprascapular nerve injury [18]. Previous studies have documented this complication in 4.2% of cases in Ekelund et al.‘s study, 2.8% in Kandziora et al.‘s trans-glenoid group, and 1.2% in Van Oostveen et al.‘s series. Importantly, most of these nerve injuries were transient, resolving spontaneously within a few months [14, 15, 17]. In our series, no cases of suprascapular nerve injury were reported in the Grand Knot group, underscoring the safety profile of this technique.

Limited shoulder external rotation range is one of the problems that can be encountered following labral repair. The selective affection of external rotation of the abducted arm may be due to the antero-inferior capsular tightness following repair [19, 20]. Notably, our series revealed significant differences in forward flexion and external rotation of abducted arm between both groups, which cannot be solely attributed to fixation techniques as this finding is reported in the literature with both suture anchors and trans-glenoid repair [11, 13, 14, 17]. We owe these differences to the fact that the preoperative range of flexion and external rotation in the Grand Knot group was significantly lower than the Suture anchor group. As the mean forward flexion was 136.5^o^ in the Grand Knot group versus 161.5^o^ in the Suture Anchor group (*P* < 0.001) and mean external rotation of abducted arm was 60.3^o^ in the Grand Knot group versus 83.8^o^ in the Suture Anchor group (*P* < 0.001) which likely influenced postoperative outcome.

In the current study, the Grand Knot technique, a modified trans-glenoid suture method is employed as a cost-effective alternative to Double-loaded suture anchors for Bankart repair. Grand Knot sutures’ costs are significantly lower (70 versus 640 USD). Considering the longer operative time expenses in these cases (50 USD) due to the need for additional fluids and medications, the Grand Knot technique remains economically advantageous (5.5-fold cost reduction). Furthermore, making the grand knot double-loaded decreases the need for more tunnels, so two tunnels can allow to repair Bankart lesion with four sutures which in turn can shorten the operative time and can lower the risk of suprascapular nerve injury when compared to the classic trans-glenoid sutures.

There were several weakness points in the current study. First, the heterogeneity of the study population in terms of pre-injury level of activity including work nature and sports activity made the comparison between both groups difficult as the functional outcome and failure rate may be related to the level of activity. The short three-year follow-up period was another weakness point. The grand knot is a novel modification of trans-glenoid repair, and the preparation of the suture block was at the beginning a time-consuming step which was not the case later on. This may have led to a significantly longer operation time in the Grand Knot group. The rehabilitation program was standardized but was carried out at different rehabilitation centers by different physiotherapists.

## Conclusion

Double-loaded grand knot technique is a surgical option for the treatment of Bankart lesions with comparable results to double-loaded anchors regarding the functional outcomes, failure, and complications rates as long as proper surgical techniques and precautions to avoid suprascapular nerve injury are considered.

## Electronic supplementary material

Below is the link to the electronic supplementary material.

Supplementary Material 1

## Data Availability

The datasets used in the current study are available from the corresponding author on reasonable request.

## Declarations

### CONSORT guidelines

Our study adheres to CONSORT guidelines.

## Abbreviations

ASES: American Shoulder and Elbow Score
ALPSA: Anterior Labral Periosteal Sleeve Avulsion
CT: Computed Tomography
MRI: Magnetic Resonance Imaging
ROM: Range of Motion
USD: United States Dollar
VAS: Visual Analog Scale
SST: Simple Shoulder Test
UCLA: University of California, Los Angeles
